# A Systematic Literature Review of Health Information Systems for Healthcare

**DOI:** 10.3390/healthcare11070959

**Published:** 2023-03-27

**Authors:** Ayogeboh Epizitone, Smangele Pretty Moyane, Israel Edem Agbehadji

**Affiliations:** 1 ICT and Society Research Group, Durban University of Technology, Durban 4001, South Africa; 2 Department of Information and Corporate Management, Durban University of Technology, Durban 4001, South Africa; 3 Centre for Transformative Agricultural and Food Systems, School of Agricultural, Earth and Environmental Sciences, University of KwaZulu-Natal, Pietermaritzburg 3209, South Africa

**Keywords:** health information system, information system, knowledge management, healthcare

## Abstract

Health information system deployment has been driven by the transformation and digitalization currently confronting healthcare. The need and potential of these systems within healthcare have been tremendously driven by the global instability that has affected several interrelated sectors. Accordingly, many research studies have reported on the inadequacies of these systems within the healthcare arena, which have distorted their potential and offerings to revolutionize healthcare. Thus, through a comprehensive review of the extant literature, this study presents a critique of the health information system for healthcare to supplement the gap created as a result of the lack of an in-depth outlook of the current health information system from a holistic slant. From the studies, the health information system was ascertained to be crucial and fundament in the drive of information and knowledge management for healthcare. Additionally, it was asserted to have transformed and shaped healthcare from its conception despite its flaws. Moreover, research has envisioned that the appraisal of the current health information system would influence its adoption and solidify its enactment within the global healthcare space, which is highly demanded.

## 1. Introduction

Health information systems (HIS) are critical systems deployed to help organizations and all stakeholders within the healthcare arena eradicate disjointed information and modernize health processes by integrating different health functions and departments across the healthcare arena for better healthcare delivery [1,2,3,4,5,6]. Over time, the HIS has transformed significantly amidst several players such as political, economic, socio-technical, and technological actors that influence the ability to afford quality healthcare services [7]. The unification of health-related processes and information systems in the healthcare arena has been realized by HIS. HIS has often been contextualized as a system that improves healthcare services’ quality by supporting management and operation processes to afford vital information and a unified process, technology, and people [7,8]. Several authors assert this disposition of HIS, alluding to its remarkable capabilities in affording seamless healthcare [9]. Haux [10] modestly chronicled HIS as a system that handles data to convey knowledge and insights in the healthcare environment. Almunawar and Anshari [7] incorporated this construed method to describe HIS to be any system within the healthcare arena that processes data and affords information and knowledge. Malaquias and Filho [11] accentuated the importance of HIS in the same light, highlighting its emergence to tackle the need to store, process, and extract information from the system data for the optimization of processes, enhancing services provided and supporting decision making.

HIS’s definition was popularized by Lippeveld [12], and reported to be an “integrated effort to collect, process, report and use health information and knowledge to influence policy-making, programme action and research”. Over the course of time, this definition has been adopted and contextualized countlessly by many authors and the World Health Organization (WHO) [3,8,13,14,15]. Although Haule, Muhanga [8] claimed the definition of HIS varies globally, in actuality, the definition has never changed from its inception, but on the contrary, it has been conceptualized over various contexts. Malaquias and Filho [11] reiterated this definition in the extant literature. These scholars affirmed HIS as “a set of interrelated components that collect, process, store and distribute information to support the decision-making process and assist in the control of health organizations” [11]. The same definition is adopted in this paper, and HIS is construed as “a system of interrelated constituents that collect, process, store and distribute data and information to support the decision-making process, assist in the control of health organizations and enhance healthcare applications”. However, it is paramount to note that HIS is broad. In many instances, the definition is of minimal relevance due to its associated incorporation with external applications related to health developments and policy making [16]. Hence, emphasis should not be placed on the definition but on its contribution to all facets of health development.

The current state of HIS is considered to be inadequate despite its numerus deployment of HIS that has been driven by its potential benefit to uplift healthcare and revolutionize its processes [17,18]. The persistence of many constraints and resistance to technology has resulted to the incapacitation of HIS in the attainment of its objectives. The extant literature reveals several challenges in different categories, such as the inadequacy of human resources and technological convergence within the healthcare [18], highlighting the evidence of limitations of HIS that restrict their utilization and deployment within the healthcare. Although several authors identified the unique disposition of HIS in integrating care and unifying the health process, these perspectives seems to be marred by the presence of barriers [17,19]. Garcia, De la Vega [17] alleged that the current HIS deployment is characterized by fragmentation, update instability, and lack of standardization that limit its potential to aid healthcare. Congruently, several authors associated the lack of awareness of HIS potential, the underuse HIS, inadequate communication network, and security and confidentiality concerns among the barriers limiting HIS [20]. Thus, the need for this paper is set forth: to uncover current and pertinent insights on HIS deployment as a concerted effort to strengthen it and augment its healthcare delivery capabilities. This paper comprehensively explores the extant literature systematically with respect to the overarching objective: to ascertain value insights pertaining to HIS holistically from literature synthesis. To achieve this goal, the following research questions are investigated: What has been the development of the HIS since its conception? How has HIS been deployed? Finally, how does HIS enable information and knowledge management in healthcare?

In this paper, an overview HIS from the extant literature in relation to the health sector is presented with associated related work. It is essential to point out that in spite of the surplus of research work conducted on health information systems, there are still many challenges confronting it within the healthcare area that necessitate the need for this study [5]. Therefore, the extant literature is explored in this paper systematically to uncover current and pertinent insights surrounding the deployment of the HIS, an integrated information system (IS) for healthcare. This paper is structured into five sections. The paper commences with an introductory background that presents the contextualization of HIS for healthcare, followed by a methodology that details the method and material used in this study. The next section, which is the discussion, presents the discourse of HIS evolution that highlights its progress to date, its structural deployment, and the information system and knowledge management within the healthcare arena as mediated by HIS. The last part of this study focuses on the conclusion that summarizes the discussion presented in this paper.

## 2. Material and Method

**Figure 1:**
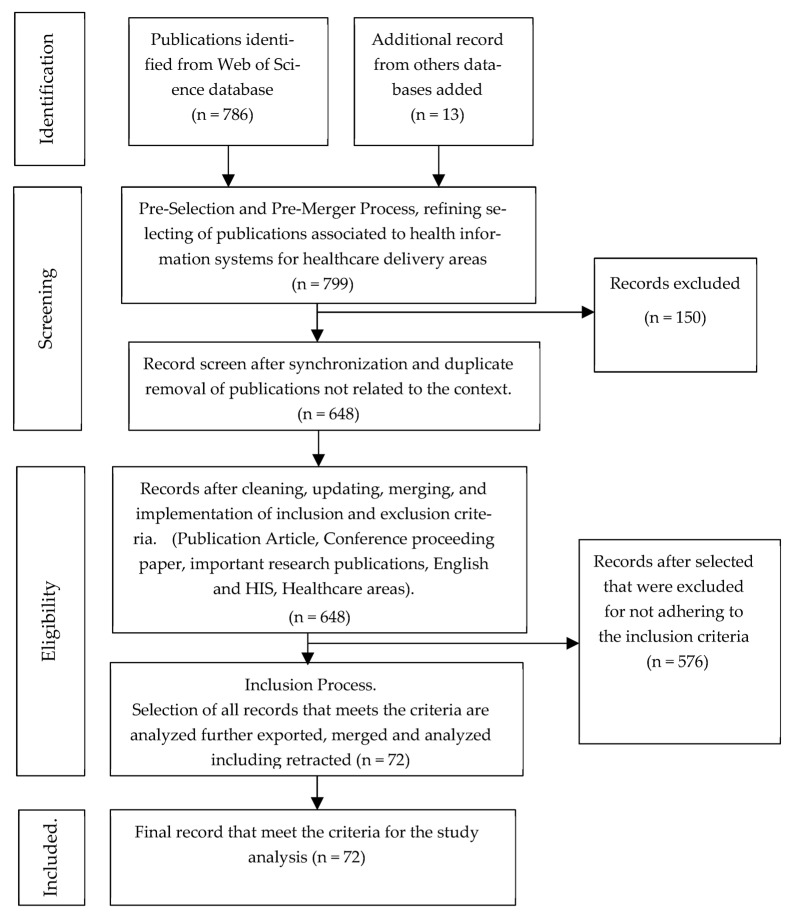
Prisma flow Statement.

In this paper, a systematic review is conducted to synthesize the extant literature and analyze the content to ascertain the value disposition of HIS in relation to healthcare delivery. Preceding this review, the used of search engines was employed to retrieve related research publications that fit the study scope and contexts. The main database used was the *Web of Science*. Other databases such as *SCOPUS* and *Google Scholar* were also used to obtain additional relevant work associated with the context. For inclusion criteria, only articles containing references to the keywords HIS, information, healthcare, and related healthcare systems were analyzed scrupulously. Research work that did not have these references, did not constitute a journal or conference-proceeding work, and were not written in the English language were excluded. Figure 1, the PRISMA flow statement, illustrates the methodological phases of this research along with the exclusion and inclusion criteria that were implemented for the study synthesis.

## 3. Discussion

### 3.1. The Evolution of Health Information Systems

The concept of enhancing healthcare applications has always been the foundation of HIS, which posits that the intercession of information systems with business processes affords better healthcare services [7,21]. According to Almunawar and Anshari [7], many determinants, such as technological, political, social and economic, have enormously influenced the nature of the healthcare industry. The technological determinant, particularly the computerized component, is thought to be deeply ingrained in the enactment and functioning of HIS. According to Panerai [16], this single attribute can be held solely responsible for HIS letdowns rather than its accomplishment.

The ownership of HIS has been contested in the literature, with some authors claiming that HIS belongs to the IT industries [22]. While IT has enabled many developments in various industries, it has also resulted in many dissatisfactions. Recently, there has been an insurgence from many industries, particularly the healthcare industries, who acknowledge the role of IT in optimizing and enhancing health initiatives but want appropriation of their integrated IS. However, according to the definition of HIS, it is presented as “a set of interconnected components that collect, process, store, and distribute information to support decision-making and aid in the control of health organizations”; thus, the disposition of HIS was established. Without bias, the development of HIS was conceived due to unavoidable changes and transformations within the global space.

A good representation and consolidation of this dispute are within the realization that there is a co-existence of different related and non-related components in a system. In this case, the HIS is an entrenched system with several features, including technologies. Panerai [16] supported this notion and theorized HIS to be broad, stating that the relevance of its definition is contextual. In the study, HIS was reiterated as any kind of “structured repository of data, information, or knowledge” that can be used to support health care delivery or promote health development [16]. Thus, maintaining a rigid definition is of minimal practical use because many HIS instances are not directly associated with health development, such as the financial and human resource modules. Moreover, several different HIS examples are categorized according to the functions they are dedicated to serving within the healthcare arena. They highlight the instances of the existence of outliers that are not regarded as the normal HIS even though they contain health determinants data, such as socioeconomic and environmental, which can be used to formulate health policies.

The development of HIS over the years has led many to believe they are solely computer technology. This notion has contributed dramatically to the misconception of the origin of HIS and the lack of peculiarity between the HIS conceptual structure and implemented HIS technology. The literature dates back the origin of HIS, which can be associated with the first record of mortality in the 18th century, revealing their existence to be 200 years or older than the invention of computers [16]. This demonstrates the emergence of digitalized HIS from the availability of commercialized episodes of “electronic medical records” EMR records in the 1970s [23]. Namageyo-Funa, Aketch [24] commended the advancement of technologies in the healthcare arena, recounting the implementation of digitalized HIS that significantly revolutionized the recording and accessing of health information. A study by Lindberg, Venkateswaran [25] highlighted an instance of HIS transition from paper based to digitally based, revealing a streamlined workflow that revolutionized health care applications in the healthcare arena. This HIS transition over the course of time has led to increased adoption of it within the health care arena. Tummers, Tekinerdogan [26] highlighted the landmark of HIS from its transition to digitalization and reported a current trend in healthcare that has now been extended with the inclusion of block chain technology within the healthcare arena. Malik, Kazi [27] assessed HIS adoption in terms of technological, organizational, human, and environmental determinants and reported a variation of different degrees of utilization. Despite these facts, the extant literature maintains the need for a resilient and sustainable HIS for health care applications within the healthcare arena at all levels [18,27,28].

**Figure 2:**
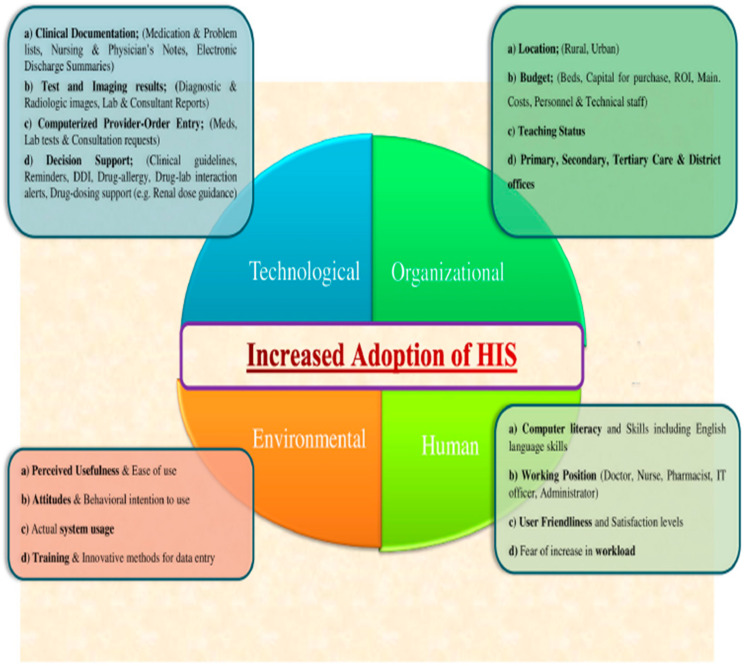
Effective health information system associations with the driving adoption determinants. Source: [27].

Figure 2 illustrates the successful adoption of HIS amidst the significant determinants of its effectiveness. From the Figure 2, the technological, organizational, human, and environmental determinants are the defining concepts along with individual sub-determinants in each domain that influence HIS adoption. At the technological level, the need for digitalization drives HIS adoption, especially for stakeholders such as clinicians and decision makers. The administrative, management, and planning functions are the driving actors within the organization level that endorse the implementation of HIS. The environmental and human determinants are more concerned with the socio-technical components that have been regarded as complex drivers for HIS adoptions. Perceptions, literacy, and usability are known forces within these categories that necessitate the adoption of HIS in many healthcare arenas.

### 3.2. HIS Structural Deployment

HIS’s unified front is geared toward assimilating and disseminating health gen to enhance healthcare delivery. HIS consists of different sub-systems that serve several actors within the healthcare arena [29]. These sub-systems are dedicated to specific tasks that perform various functions such as civil registrations, disease surveillance, outbreak notices, interventions, and health information sharing within the healthcare arena. It also supports and links many functions and activities within the healthcare environment, such as recording various data and information for stakeholders, scheduling, billing, and managing. Stakeholders are furnished with health information from diverse HIS scenarios. These include but are not limited to information systems for hospitals and patients, health institution systems, and Internet information systems. Sligo, Gauld [30] regarded HIS as a panacea within the healthcare ground that improves health care applications. Despite all the limitless capabilities of HIS, it has been reported to be asymmetrical, lacking interactions within subsystems [1,18]. Many decision making methods and policies rely on good health information [31]. According to Suresh and Singh [32], the HIS enables stakeholders such as the government and all other players in the healthcare arena to have access to health information, which influences the delivery of healthcare. The sundry literature further reveals accurate health information to be the foundation of decision making and highlights the decisive role of the human constituent [29,31,33,34].

**Figure 3:**
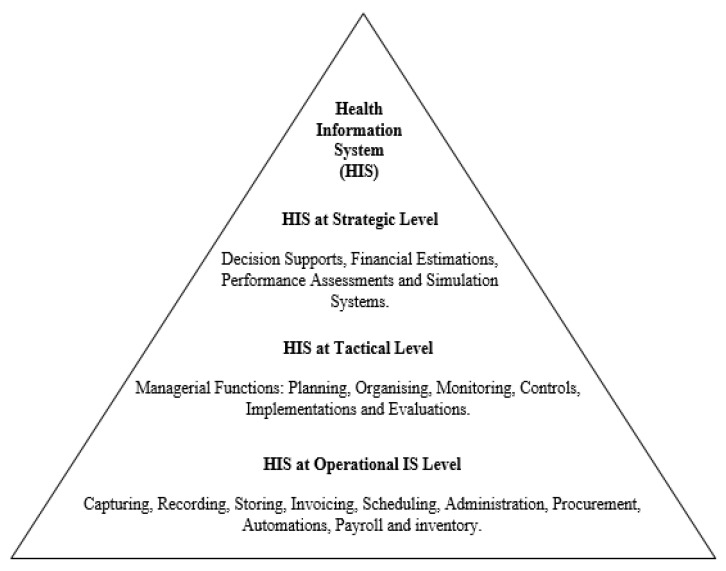
Levels of HIS deployment: source authors.

Furthermore, HIS can be classified into two cogs in today’s era: the computer-related constituent that employs ICT-related tools and the non-computer component, which both operate at different levels. These levels include strategic, tactical, and operational. The deployment of HIS at the strategic level offers intelligence functions such as intelligent decision support, financial estimation, performance assessment, and simulation systems [3,35]. At the tactical level, managerial functions are performed within the system, while at the operational level, functions including recording, invoicing, scheduling, administrative, procurement, automation, and even payroll are carried out. Figure 3 shows the three levels within the healthcare system where HIS deployment is utilized.

### 3.3. Health Information Systems Benefits

HIS, as an interrelated system, houses several core processes and branches in the healthcare arena, affording many benefits. Among these are the ease of access to patients and medical records, reduction of costs and time, and evidence-based health policies and interventions [8,21,36,37,38]. Several authors revealed the benefits of HIS to be widely known and influential within the healthcare domain [38]. Furthermore, many health organizations are drawn to HIS because of these numerous advantages [22,39]. Moreover, investment in HIS has enabled effective decision making, real-time comprehensive health information for quality health care applications, effective policies in the healthcare arena, scaled-up monitoring and evaluation, health innovations, resource allocations, surveillance services, and enhanced governance and accountability [36,40,41,42]. Ideally, HIS is pertinent for data, information, and broad knowledge sharing in the healthcare environment. HIS critical features are now cherished due to their incorporation with diverse technology [16,43]. The extant literature reveals the role of HIS to extend beyond its reimbursement. Table 1 presents a summarized extract of various HIS benefits as captured in the literature and some of its core enabling components or instances. 

### 3.4. Information System and Knowledge Management in the Healthcare Arena

The presence of modernized information systems (IS) in the healthcare arena is alleged by scholars to be a congested domain that seldom fosters stakeholders’ multifaceted and disputed relationships [48]. On the other hand, it is believed that a significant amount of newly acquired knowledge in the field of healthcare is required for the improvement of health care [49]. Ascertaining and establishing the role of IS and knowledge management is an important step in the development of HIS for healthcare. Flora, Margaret [5] posited that efficient IS and data usage are crucial for an effective healthcare system. Bernardi [50] alleged that the underpinning inkling of a “robust and efficient” HIS enables healthcare stakeholders such as managers and providers to leverage health information to commendably plan and regulate healthcare, which could result in enhanced survival rates. As a result, it is imperative to ground these ideas within the context of the healthcare industry to provide a foundation for developing a robust and sustainable HIS for use in the context of health care applications.

#### 3.4.1. Information System

The assimilation and dissimilation of health information and data within the healthcare system is an important task that influences healthcare outcome. Within the healthcare setting, IS plays a significant role in the assimilation and dissimilation of health information needed by healthcare stakeholders. Many continents endorse the deployment of IS mainly to consolidate mutable information from different sources within the systems. The primary objective for these systems’ deployment has been centered on bringing together unique and different components such as institutions, people, processes, and technology in the system under one umbrella [5,51]. An overview of the extant literature reveals that this has rarely been easy, as integration within this system has always been difficult in many contexts. In the context of HIS, many reported the integration phenomena to be problematic, attributing this to the global transformation within the healthcare arena [52,53]. This revolution, coupled with the advancement of the healthcare arena, has resulted in the need for robust allied health IS systems that incorporates different IS and information technology [5,22]. These allied health information systems are necessary to consolidate independent information systems within their healthcare arena use to enhance healthcare applications [54,55]. Organizations in the healthcare arena expect these systems to be sustainable and resilient; however, in order to satisfy these requirements, an integrated information system is needed to unify all independent, agile, and flexible health IS to mitigate challenges for HIS [56].

An aligned HIS that is allied is essential, as it supports health information networks (HIN) that subsequently enhance and improve healthcare applications [44,57]. Thus, many organizations within the healthcare settings are fine-tuning their HIS to be resilient and sustainable. However, the realization of a robust information system within the healthcare arena is challenging and depends on the flow of information as a crucial constituent for suave and efficient functioning [58,59].

#### 3.4.2. Knowledge Management

The process of constructing value and generating a maintainable edge for an industry with capitalization on building, communicating, and knowledge applications procedures to realize set aspirations is denoted as knowledge management [60]. The literature reveals knowledge management as an important contributor to organizational performance through its knowledge-sharing capabilities [61]. In the healthcare industry, there is a high demand for knowledge to enhance healthcare applications [49,62]. Several studies reported that the deployment of knowledge management in the healthcare arena is set to enhance healthcare treatment effectiveness [49,58,61]. Many stakeholders such as governments, World Health Organization (WHO), and healthcare workers rely on the management of healthcare knowledge to complement healthcare applications. According to Kim, Newby-Bennett [61], the focus of knowledge management is to efficaciously expedite knowledge sharing. However, integrating knowledge from different sources is challenging and requires an enabler [61].

The HIS is an indispensable enabler of health knowledge generated from amalgamated health information within the healthcare arena [63,64,65]. Dixon, McGowan [66] asserted that efficacious modifications in the healthcare arena are made possible by knowledge codification and collaboration from information technologies. Similarly, some authors have pinpointed information and communication technologies within the healthcare arena to be a major determinant in the attainment of a sustainable health system development [58]. The knowledge management relationship with HIS is considered complementary and balanced, as it enables the availability of knowledge that can be shared. The importance of knowledge management is relevant for the realization of an enhanced healthcare application via HIS. Soltysik-Piorunkiewicz and Morawiec [58] claimed that the information society effectively uses HIS as an information system for management, patient knowledge, health knowledge, healthcare unit knowledge, and drug knowledge. The authors herein demonstrated how HIS facilitates knowledge management in the healthcare sector to improve healthcare applications.

The role of HIS as an integrated IS and key enabler of healthcare knowledge management highlights its potential within the healthcare arena. From the conception of HIS and the records of its evolution, significant achievements have been attained that are demonstrated at different levels of its structural deployment. HIS deployment in several settings of healthcare have positively influenced clinical processes and patients’ outcomes [17]. Globally, the need for HIS within the healthcare system is critical in the enhancement of healthcare. Many healthcare actions are dependent on the use of HIS [67,68,69]. This demand is substantiated by the offerings of HIS in tackling the transformation and digitalization confronting the healthcare system. However, despite the need for HIS and its potential within healthcare, several barriers limit its optimization. Some authors posited the role and involvement of healthcare professionals such as physicians to be important measure that is paramount to decreasing the technical and personal barriers sabotaging HIS deployment [20]. Nonetheless, the design of HIS is accentuated on augmenting health and is considered to be lagging behind in attaining quality healthcare [70].

Although there are equal blessings as well as challenges with HIS deployment, this study appraisal of HIS highlights its capabilities and attributes that enhance healthcare in many ways. From its conception, HIS has evolved significantly to enable the digitalization of many healthcare processes. Its deployment structurally has facilitated many healthcare applications at all levels within the health system where it has been implemented. Many benefits such as ease of access to medical records, cost reduction, data and information management, precision medicine, and autonomous and intelligent decisions have been enabled by HIS deployment. Primarily, HIS is the core enabler of the healthcare information system and knowledge management within the healthcare arena. Ascertaining the attributes and development of HIS is a paramount to driving its implementation and realizing its potential. Many deployments of HIS can be anchored on this study as a reference for planning and executing HIS implementation. The extant literature points out the need for the role of technology such HIS to be ascertained, as little is known in this regard, which as a result has adversely influenced healthcare coordination [19]. Additionally, among the barriers of HIS, the presence of inadequate planning that fails to cater to the needs of those adopting it hinders the optimization of these systems within the healthcare arena [71]. Cawthon, Mion [72] associated the lack of health literacy incorporation in deployed HIS to increased cost and poorer health outcomes. Hence, the insight from this study can be incorporated and associated with HIS initiatives to mitigate these issues. Thus, the findings of this study can be employed to strategize HIS deployment and plans as well as augment its potential to enhance healthcare. Furthermore, the competency of healthcare stakeholders such as patients can be enhanced with the findings of this study that accentuate the holistic representation of HIS in the dissimilation and assimilation of health data and information.

## 4. Conclusions

In the healthcare information and knowledge arena, assimilation and dissemination is a facet that influences healthcare delivery. The conception and evolution of HIS has positioned this system within the healthcare arena to arbitrate information interchange for its stakeholders. HIS deployment within healthcare has not only enabled information and knowledge management, but it has also enabled and driven many healthcare agendas and continues to maintain a solidified presence within the healthcare space. However, its deployment and enactment globally has been marred and plagued with several challenges that hinder its optimization and defeat its purpose. Phenomena such as the occurrences of pandemics such as COVID-19, which are uncertain, and the advancement of technology that cannot be controlled have caused disputed gradients regarding the positioning of HIS. These phenomena have not only influenced the adoption of HIS but have also limited its ability to be fully utilized. Although much research on HIS has been conducted, the presence of these phenomena and many other inherent challenges such as fragmentation and cost still maintain a constant, prominent presence, which has led to the need for this study.

Consequently, the starting point for this study was to provide insight and expertise regarding the discourse of HIS for healthcare applications. This paper presents current and pertinent insights regarding the deployment of the HIS that, when adopted, can positively aid its employment. This paper investigated the existing HIS literature to accomplish the objective set forth in the introduction. This study’s synthesis derived key insights relevant to the holistic view of HIS through a thorough systematic review of the various extant literature on HIS and healthcare. According to the study’s findings, HIS are critical and foundational in the drive of information and knowledge management for healthcare. The contribution of HIS to healthcare has been and continues to be groundbreaking since its conception and through its consequent evolution. Nevertheless, despite the presence of some limitations that are external and inherent, it is claimed to have transformed and changed healthcare from the start. Similarly, the evaluation of the current HIS is expected to impact its adoption and strengthen its implementation within the global healthcare space, which is greatly desired. These findings are of great importance to the healthcare stakeholders that directly and indirect interact with HIS. Additionally, scholars and healthcare researchers can benefit from this study by incorporating the findings in future works that plan HIS for healthcare.

## Figures and Tables

**Table 1:** HIS core enabling components and its benefits.

Source: Authors	Core Enabling HIS Components	Benefits
Malaquias and Filho [11]	Health ER eHealth mHealth	Ease of access to patient and medical information from records; Cost reduction; Enhance efficiency in patients’ data recovery and management; Enable stakeholders’ health information centralization and remote access.
Ammenwerth, Duftschmid [44]	eHealth	Upsurge in care efficacy and quality and condensed costs for clinical services; Lessen the health care system’s administrative costs; Facilitates novel models of health care delivery.
Tummers, Tobi [45]	HIS	Patient information management; Enable communication within the healthcare arena; Afford high-quality and efficient care.
Steil, Finas [46]	HIS	Enable inter- and multidisciplinary collaboration between humans and machines; Afford autonomous and intelligent decision capabilities for health care applications.
Nyangena, Rajgopal [43]	HIS	Enable seamless information exchange within the healthcare arena.
Sik, Aydinoglu [47]	HIS	Support precision medicine approaches and decision support.

## Data Availability

Not applicable.

## References

[1] Sahay S., Nielsen P., Latifov M. (2018). Grand challenges of public health: How can health information systems support facing them?. Health Policy Technol..

[2] English R., Masilela T., Barron P., Schonfeldt A. (2011). Health information systems in South Africa. S. Afr. Health Rev..

[3] Bagayoko C.O., Tchuente J., Traoré D., Moukoumbi Lipenguet G., Ondzigue Mbenga R., Koumamba A.P., Ondjani M.C., Ndjeli O.L., Gagnon M.P. (2020). Implementation of a national electronic health information system in Gabon: A survey of healthcare providers’ perceptions. BMC Med. Inform. Decis. Mak..

[4] Berrueta M., Bardach A., Ciaponni A., Xiong X., Stergachis A., Zaraa S., Buekens P. (2020). Maternal and neonatal data collection systems in low- and middle-income countries: Scoping review protocol. Gates Open Res..

[5] Flora O.C., Margaret K., Dan K. (2017). Perspectives on utilization of community based health information systems in Western Kenya. Pan Afr. Med. J..

[6] Rachmani E., Lin M.C., Hsu C.Y., Jumanto J., Iqbal U., Shidik G.F., Noersasongko E. (2020). The implementation of an integrated e-leprosy framework in a leprosy control program at primary health care centers in Indonesia. Int. J. Med. Inform..

[7] Almunawar M.N., Anshari M. (2012). Health information systems (HIS): Concept and technology. arXiv.

[8] Haule C.D., Muhanga M., Ngowi E. (2022). The what, why, and how of health information systems: A systematic review. Sub Sahar. J. Soc. Sci. Humanit..

[9] Epizitone A., Moyane S.P., Agbehadji I.E. (2022). Health Information System and Health Care Applications Performance in the Healthcare Arena: A Bibliometric Analysis. Healthcare.

[10] Haux R. (2006). Health information systems–past, present, future. Int. J. Med. Inform..

[11] Malaquias R.S., Filho I.M.B., Gervasi O., Murgante B., Misra S., Garau C., Blecic I., Taniar D., Apduhan B.O., Rocha A.M., Tarantino E., Torre C.M. (2021). Middleware for Healthcare Systems: A Systematic Mapping. Proceedings of the 21st International Conference on Computational Science and Its Applications, ICCSA 2021.

[12] Lippeveld T. Routine health information systems: The glue of a unified health system. Proceedings of the Keynote address at the Workshop on Issues and Innovation in Routine Health Information in Developing Countries.

[13] AbouZahr C., Boerma T. (2005). Health information systems: The foundations of public health. Bull. World Health Organ..

[14] Bogaert P., Van Oyen H. (2017). An integrated and sustainable EU health information system: National public health institutes’ needs and possible benefits. Arch. Public Health.

[15] Bogaert P., van Oers H., Van Oyen H. (2018). Towards a sustainable EU health information system infrastructure: A consensus driven approach. Health Policy.

[16] Panerai R. (2014). Health Information Systems.

[17] Garcia A.P., De la Vega S.F., Mercado S.P. (2022). Health Information Systems for Older Persons in Select Government Tertiary Hospitals and Health Centers in the Philippines: Cross-sectional Study. J. Med. Internet Res..

[18] Epizitone A. (2022). Framework to Develop a Resilient and Sustainable Integrated Information System for Health Care Applications: A Review. Int. J. Adv. Comput. Sci. Appl. (IJACSA).

[19] Walcott-Bryant A., Ogallo W., Remy S.L., Tryon K., Shena W., Bosker-Kibacha M. (2021). Addressing Care Continuity and Quality Challenges in the Management of Hypertension: Case Study of the Private Health Care Sector in Kenya. J. Med. Internet Res..

[20] Malekzadeh S., Hashemi N., Sheikhtaheri A., Hashemi N.S. (2018). Barriers for Implementation and Use of Health Information Systems from the Physicians’ Perspectives. Stud. Health Technol. Inform..

[21] Tossy T. (2014). Major challenges and constraint of integrating health information systems in african countries: A Namibian experience. Int. J. Inf. Commun. Technol..

[22] Vaganova E., Ishchuk T., Zemtsov A., Zhdanov D. Health Information Systems: Background and Trends of Development Worldwide and in Russia. Proceedings of the 10th International Joint Conference on Biomedical Engineering Systems and Technologies-Volume 5: HEALTHINF, (BIOSTEC 2017).

[23] Thomas J., Carlson R., Cawley M., Yuan Q., Fleming V., Yu F. (2022). The Gap Between Technology and Ethics, Especially in Low-and Middle-Income Country Health Information Systems: A Bibliometric Study. Stud. Health Technol. Inform..

[24] Namageyo-Funa A., Aketch M., Tabu C., MacNeil A., Bloland P. (2018). Assessment of select electronic health information systems that support immunization data capture—Kenya, 2017. BMC Health Serv. Res..

[25] Lindberg M.H., Venkateswaran M., Abu Khader K., Awwad T., Ghanem B., Hijaz T., Morkrid K., Froen J.F. (2019). eRegTime, Efficiency of Health Information Management Using an Electronic Registry for Maternal and Child Health: Protocol for a Time-Motion Study in a Cluster Randomized Trial. JMIR Res. Protoc..

[26] Tummers J., Tekinerdogan B., Tobi H., Catal C., Schalk B. (2021). Obstacles and features of health information systems: A systematic literature review. Comput. Biol. Med..

[27] Malik M., Kazi A.F., Hussain A. (2021). Adoption of health technologies for effective health information system: Need of the hour for Pakistan. PLoS ONE.

[28] De Carvalho Junior M.A., Bandiera-Paiva P. (2018). Health Information System Role-Based Access Control Current Security Trends and Challenges. J. Healthc Eng..

[29] Taye G. (2021). Improving health care services through enhanced Health Information System: Human capacity development Model. Ethiop. J. Health Dev..

[30] Sligo J., Gauld R., Roberts V., Villa L. (2017). A literature review for large-scale health information system project planning, implementation and evaluation. Int. J. Med. Inform..

[31] Bosch-Capblanch X., Oyo-Ita A., Muloliwa A.M., Yapi R.B., Auer C., Samba M., Gajewski S., Ross A., Krause L.K., Ekpenyong N. (2021). Does an innovative paper-based health information system (PHISICC) improve data quality and use in primary healthcare? Protocol of a multicountry, cluster randomised controlled trial in sub-Saharan African rural settings. BMJ Open.

[32] Suresh L., Singh S.N. (2014). Studies in ICT and Health Information System. Int. J. Inf. Libr. Soc..

[33] Isleyen F., Ulgu M.M. (2020). Data Transfer Model for HIS and Developers Opinions in Turkey. Stud. Health Technol. Inform..

[34] Jeffery C., Pagano M., Hemingway J., Valadez J.J. (2018). Hybrid prevalence estimation: Method to improve intervention coverage estimations. Proc. Natl. Acad. Sci. USA.

[35] Sawadogo-Lewis T., Keita Y., Wilson E., Sawadogo S., Téréra I., Sangho H., Munos M. (2021). Can We Use Routine Data for Strategic Decision Making? A Time Trend Comparison Between Survey and Routine Data in Mali. Glob. Health Sci. Pract..

[36] Kpobi L., Swartz L., Ofori-Atta A.L. (2018). Challenges in the use of the mental health information system in a resource-limited setting: Lessons from Ghana. BMC Health Serv. Res..

[37] Feteira-Santos R., Camarinha C., Nobre M.D., Elias C., Bacelar-Nicolau L., Costa A.S., Furtado C., Nogueira P.J. (2022). Improving morbidity information in Portugal: Evidence from data linkage of COVID-19 cases surveillance and mortality systems. Int. J. Med. Inform..

[38] Ker J.I., Wang Y.C., Hajli N. (2018). Examining the impact of health information systems on healthcare service improvement: The case of reducing in patient-flow delays in a US hospital. Technol. Forecast. Soc. Chang..

[39] Alahmar A., AlMousa M., Benlamri R. (2022). Automated clinical pathway standardization using SNOMED CT- based semantic relatedness. Digital Health.

[40] Krasuska M., Williams R., Sheikh A., Franklin B., Hinder S., TheNguyen H., Lane W., Mozaffar H., Mason K., Eason S. (2021). Driving digital health transformation in hospitals: A formative qualitative evaluation of the English Global Digital Exemplar programme. BMJ Health Care Inform..

[41] Dunn T.J., Browne A., Haworth S., Wurie F., Campos-Matos I. (2021). Service Evaluation of the English Refugee Health Information System: Considerations and Recommendations for Effective Resettlement. Int. J. Environ. Res. Public Health.

[42] See E.J., Bello A.K., Levin A., Lunney M., Osman M.A., Ye F., Ashuntantang G.E., Bellorin-Font E., Benghanem Gharbi M., Davison S. (2022). Availability, coverage, and scope of health information systems for kidney care across world countries and regions. Nephrol. Dial. Transplant..

[43] Nyangena J., Rajgopal R., Ombech E.A., Oloo E., Luchetu H., Wambugu S., Kamau O., Nzioka C., Gwer S., Ndirangu M.N. (2021). Maturity assessment of Kenya’s health information system interoperability readiness. BMJ Health Care Inform..

[44] Ammenwerth E., Duftschmid G., Al-Hamdan Z., Bawadi H., Cheung N.T., Cho K.H., Goldfarb G., Gulkesen K.H., Harel N., Kimura M. (2020). International Comparison of Six Basic eHealth Indicators Across 14 Countries: An eHealth Benchmarking Study. Methods Inf. Med..

[45] Tummers J., Tobi H., Schalk B., Tekinerdogan B., Leusink G. (2021). State of the practice of health information systems: A survey study amongst health care professionals in intellectual disability care. BMC Health Serv. Res..

[46] Steil J., Finas D., Beck S., Manzeschke A., Haux R. (2019). Robotic Systems in Operating Theaters: New Forms of Team-Machine Interaction in Health Care On Challenges for Health Information Systems on Adequately Considering Hybrid Action of Humans and Machines. Methods Inf. Med..

[47] Sik A.S., Aydinoglu A.U., Son Y.A. (2021). Assessing the readiness of Turkish health information systems for integrating genetic/genomic patient data: System architecture and available terminologies, legislative, and protection of personal data. Health Policy.

[48] Bernardi R., Constantinides P., Nandhakumar J. (2017). Challenging Dominant Frames in Policies for IS Innovation in Healthcare through Rhetorical Strategies. J. Assoc. Inf. Syst..

[49] Liu G., Tsui E., Kianto A. (2022). An emerging knowledge management framework adopted by healthcare workers in China to combat COVID-19. Knowl. Process Manag..

[50] Bernardi R. (2017). Health Information Systems and Accountability in Kenya: A Structuration Theory Perspective. J. Assoc. Inf. Syst..

[51] Epizitone A. (2021). Critical Success Factors within an Enterprise Resource Planning System Implementation Designed to Support Financial Functions of a Public Higher Education Institution. Master’s Thesis.

[52] Ostern N., Perscheid G., Reelitz C., Moormann J. (2021). Keeping pace with the healthcare transformation: A literature review and research agenda for a new decade of health information systems research. Electron. Mark..

[53] Farnham A., Utzinger J., Kulinkina A.V., Winkler M.S. (2020). Using district health information to monitor sustainable development. Bull. World Health Organ..

[54] Faujdar D.S., Sahay S., Singh T., Kaur M., Kumar R. (2020). Field testing of a digital health information system for primary health care: A quasi-experimental study from India. Int. J. Med. Inform..

[55] Jabareen H., Khader Y., Taweel A. (2020). Health information systems in Jordan and Palestine: The need for health informatics training. East. Mediterr. Health J..

[56] Ayabakan S., Bardhan I., Zheng Z., Kirksey K. (2017). The Impact of Health Information Sharing on Duplicate Testing. MIS Q..

[57] Mayer F., Faglioni L., Agabiti N., Fenu S., Buccisano F., Latagliata R., Ricci R., Spiriti M.A.A., Tatarelli C., Breccia M. (2017). A Population-Based Study on Myelodysplastic Syndromes in the Lazio Region (Italy), Medical Miscoding and 11-Year Mortality Follow-Up: The Gruppo Romano-Laziale Mielodisplasie Experience of Retrospective Multicentric Registry. Mediterr. J. Hematol. Infect. Dis..

[58] Soltysik-Piorunkiewicz A., Morawiec P. (2022). The Sustainable e-Health System Development in COVID 19 Pandemic–The Theoretical Studies of Knowledge Management Systems and Practical Polish Healthcare Experience. J. e-Health Manag..

[59] Seo K., Kim H.N., Kim H. (2019). Current Status of the Adoption, Utilization and Helpfulness of Health Information Systems in Korea. Int. J. Environ. Res. Public Health.

[60] Mahendrawathi E. (2015). Knowledge management support for enterprise resource planning implementation. Procedia Comput. Sci..

[61] Kim Y.M., Newby-Bennett D., Song H.J. (2012). Knowledge sharing and institutionalism in the healthcare industry. J. Knowl. Manag..

[62] Nwankwo B., Sambo M.N. (2020). Effect of Training on Knowledge and Attitude of Health Care Workers towards Health Management Information System in Primary Health Centres in Northwest Nigeria. West Afr. J. Med..

[63] Khader Y., Jabareen H., Alzyoud S., Awad S., Rumeileh N.A., Manasrah N., Mudallal R., Taweel A. Perception and acceptance of health informatics learning among health-related students in Jordan and Palestine. Proceedings of the 2018 IEEE/ACS 15th International Conference on Computer Systems and Applications (AICCSA).

[64] Benis A., Harel N., Barak Barkan R., Srulovici E., Key C. (2018). Patterns of Patients’ Interactions With a Health Care Organization and Their Impacts on Health Quality Measurements: Protocol for a Retrospective Cohort Study. JMIR Res. Protoc..

[65] Delnord M., Abboud L.A., Costa C., Van Oyen H. (2021). Developing a tool to monitor knowledge translation in the health system: Results from an international Delphi study. Eur. J. Public Health.

[66] Dixon B.E., McGowan J.J., Cravens G.D. (2009). Knowledge sharing using codification and collaboration technologies to improve health care: Lessons from the public sector. Knowl. Manag. Res. Pract..

[67] See E.J., Alrukhaimi M., Ashuntantang G.E., Bello A.K., Bellorin-Font E., Gharbi M.B., Braam B., Feehally J., Harris D.C., Jha V. (2018). Global coverage of health information systems for kidney disease: Availability, challenges, and opportunitiesfor development. Kidney Int. Suppl..

[68] Vicente E., Ruiz de Sabando A., García F., Gastón I., Ardanaz E., Ramos-Arroyo M.A. (2021). Validation of diagnostic codes and epidemiologic trends of Huntington disease: A population-based study in Navarre, Spain. Orphanet J. Rare Dis..

[69] Colais P., Agabiti N., Davoli M., Buttari F., Centonze D., De Fino C., Di Folco M., Filippini G., Francia A., Galgani S. (2017). Identifying Relapses in Multiple Sclerosis Patients through Administrative Data: A Validation Study in the Lazio Region, Italy. Neuroepidemiology.

[70] De Sanjose S., Tsu V.D. (2019). Prevention of cervical and breast cancer mortality in low- and middle-income countries: A window of opportunity. Int. J. Womens Health.

[71] Aung E., Whittaker M. (2013). Preparing routine health information systems for immediate health responses to disasters. Health Policy Plan..

[72] Cawthon C., Mion L.C., Willens D.E., Roumie C.L., Kripalani S. (2014). Implementing routine health literacy assessment in hospital and primary care patients. Jt. Comm. J. Qual. Patient Saf..

